# Incidence of central precocious puberty declines to pre-pandemic levels post COVID-19 pandemic increase: single-center retrospective review in the United States

**DOI:** 10.3389/fped.2024.1352295

**Published:** 2024-03-13

**Authors:** Marcela Vargas Trujillo, Tiranun Rungvivatjarus, Karen O. Klein

**Affiliations:** ^1^ Department of Pediatrics, University of California San Diego, San Diego, CA, United States; ^2^ Rady Children's Hospital, San Diego, CA, United States

**Keywords:** CPP, COVID-19, pandemic, incidence, puberty

## Abstract

**Background and aim of the study:**

We previously published the increased frequency of new CPP cases during the Covid-19 pandemic in our pediatric endocrinology clinic at Rady Children's Hospital in San Diego, CA, US. We conducted this follow-up study to examine the incidence of new CPP cases requiring treatment with GnRH agonist (GnRHa) in our clinic during 2 years post-pandemic.

**Methods:**

We performed a retrospective comparison of the number of visits of children newly diagnosed with CPP treated with GnRHa during the 2 years following the first year of Covid-19 pandemic (5/2021–7/2023). We evaluated clinical and bone maturation data as well as differences in timing from diagnosis to onset of treatment.

**Results:**

We previously reported in the pre-Covid year, 28 children (1 boy, 27 girls) treated with GnRHa for CPP out of 2,340 new endocrinology visits (1.2% of patients seen). During Covid-19 year, 64 children (7 boys, 57 girls) were treated out of 2,261 new visits (2.8%). The incidence of new CPP cases requiring GnRHa during the pandemic more than doubled compared to pre-pandemic. In the first year post-year 1 of the pandemic (5/2021–4/2022), 46 children (3 boys, 40 girls) started treatment with GnRHa for CPP out of 2,595 new endocrinology visits (1.6% of patients seen). During the second follow-up year (5/2022–4/2023), 22 children (4 boys, 18 girls) started treatment with GnRHa for CPP out of 2,676 new endocrinology visits (0.8% of patients seen). Age at onset of treatment, degree of bone age (BA) advancement, time from diagnosis to onset of treatment, and changes in BMI during the pandemic were not different from pre-pandemic or post-pandemic.

**Conclusions:**

CPP cases requiring GnRHa treatment significantly increased during the first year of the Covid-19 pandemic and then decreased each year post-pandemic. This was not related to BMI, age at diagnosis, degree of bone age advancement, or time from diagnosis to onset of treatment as all these factors have been similar during pre-pandemic, pandemic, and post-pandemic years. It is reasonable that the postulated hypotheses published regarding the increase during the pandemic would resolve post-pandemic.

## Introduction

We and others previously reported an increase in cases of CPP during the first year of Covid-19 (1–9). To evaluate if this trend continued further into or post-pandemic, we conducted this follow-up study to assess whether the incidence of CPP cases in years post-pandemic continued at the increased rate or returned to pre-pandemic rates. Despite ample evidence that CPP cases increased during the pandemic, the mechanisms for this change in incidence are not yet well understood and many hypotheses remain unexplained. Most hypotheses link the increased incidence to either a direct effect of SARS-CoV-2 infection, or to factors associated with the change in lifestyle and associated emotional and physical changes induced by social isolation during the pandemic. Understanding trends post-Covid-19, may bring insight into those hypotheses and help target areas for research.

## Methods

### Study design and setting

We performed a single-center retrospective comparison of the incidence of newly diagnosed children with CPP requiring GnRHa treatment pre-Covid-19 year (5/2018–4/2019), during the Covid-19 pandemic (5/2020–4/2021) and 2 years post-Covid-19 (5/2021–4/2022, and 5/2022–4/2023). The study was conducted at a free-standing, tertiary care academic children's hospital and pediatric endocrinology specialty outpatient clinic in San Diego, California. Patients with suspected CPP are referred to endocrinology clinic by general pediatricians and other subspecialists practicing in the San Diego County and surrounding areas. Subjects were identified by electronic medical record (EMR) query (Epic Systems Corporation, Verona, Wisconsin). A new diagnosis of CPP was defined as having all of the following: (1) at least 1 endocrinology clinic visit associated with one of the following 4 CPP ICD codes: early puberty, premature thelarche, precocious puberty, or central precious puberty; (2) chronological age <8 years for girls and <9 years for boys at onset of symptoms; (3) a random luteinizing hormone (LH) level >0.3 IU/L (10), or a GnRH-stimulated peak LH level >5 IU/L (11), or a GnRH-stimulated estradiol >40 pg/ml in girls (12, 13) or testosterone >30 ng/dl in boys (13). GnRHa stimulation tests were performed by administering aqueous leuprolide acetate subcutaneously, at a standard dose of 20 mcg/kg (maximum dose 500 mcg). Blood samples were obtained at 1 h for measurement of LH level and at 18–24 h for measurement of estradiol in girls or testosterone in boys. In addition, only patients who received GnRHa treatment were included to eliminate the potential for borderline cases meeting diagnostic criteria but not deemed necessary to treat. Date of diagnosis was defined by date of confirmatory blood test. Patient characteristics and outcome variables were obtained by EMR query and manual chart review (KK, MVT). Bone age assessment was done according to the atlas of Greulich and Pyle (14) and determined by the patient's endocrinologist. The University of California San Diego (UCSD) Institutional Review Board approved this study, and a waiver of informed consent was granted for the collection.

Descriptive analysis used means ± standard deviation (SD). Chi-square was used for comparing proportions of newly diagnosed patients with CPP during different years. Two-tailed Student T-tests were used to compare variables of retrospective data.

The primary outcome measure was the proportion of endocrinology visits associated with new CPP diagnosis and GnRHa treatment during the 2 years post an initial year of Covid-19 pandemic compared to the pre-pandemic and pandemic period. We evaluated bone age (BA), BMI, time from diagnosis to GnRHa order, and time from GnRHa order to first day of treatment. GnRHa included leuprolide, triptorelin, and histrelin. First day of GnRHa treatment was captured by the date of the first endocrinology nurse visit for GnRHa administration or day of insertion for the patient receiving histrelin implant.

### Analysis

Between groups. We obtained the data regarding Covid-19 cases per month in California and in San Diego County from Government data (15, 16) for children under 18 years of age. San Diego infection rates paralleled those of California, so California data was used for more robust statistics and divided by 3,000 for visual scaling.

## Results

### Incidence of patients diagnosed with CPP and treated with GnRHa

There were 2,340 new patients seen in our endocrinology clinic during pre-Covid-19 year (5/2019–4/2019), including all chief complaints. Of these, 28 children (1 boy, 27 girls) were diagnosed with CPP and treated with GnRHa (1.2% of patients seen). During Covid-19 year (5/2020–4/2021), 64 children (7 boys, 57 girls) were diagnosed with CPP and treated with GnRHa, out of 2,261 new visits (2.8% of patients seen). In the first year post-year 1 of the pandemic (5/2021–4/2022), 43 children (3 boys, 40 girls) started treatment with GnRHa for CPP out of 2,595 new endocrinology visits (1.6% of patients seen). During the following year (5/2022–4/2023), 22 children (4 boys, 18 girls) started treatment with GnRHa for CPP out of 2,676 new endocrinology visits (0.8% of patients seen).

The incidence of new CPP cases requiring GnRHa more than doubled during Covid compared to the pre-Covid years (*p* < 0.01, Chi Square) and decreased to pre-covid rates over 2 subsequent years.

### Patient demographics

Onset of puberty was less than 8 years for all girls and less than 9 years for all boys. The mean age at diagnosis was 7.1 ± 2.1 (1.8–10.0) years in pre-Covid-19 years, 7.6 ± 1.43 (1.5–10.5) (7.3 ± 1.4 for girls, 6.5 ± 2.4 for boys) years during Covid-19 year, and significantly higher at 8.12 ± 1.4 (2.6–10.6) (8.0 ± 1.3 for girls, 9.5 ± 0.8 for boys) years over 2 years post covid (*p* = 0.0004 and *p* = 0.01 for girls, *p* < 0.05 for boys). BMI in pre-Covid-19 years was 17.6 ± 2.8 kg/m^2^ (12.7–25.5), 18.2 ± 3.5 kg/m^2^ (11.4–29.4) during Covid-19, and 18.5 ± 2.5 kg/m^2^ (13.6–29.3) over 2 years post covid (similar for girls and boys. Bone age at diagnosis was 9.5 ± 2.6 y (1.6–13.0) in the pre-Covid-19 year, 9.5 ± 1.7 y (2.5–12.6) in the Covid-19 year, and 10.6 ± 1.4 y (6.8–13) (10.4 ± 1.3 for girls, 11.9 ± 1.2 for boys) over the 2 years post-Covid-19, consistent with increasing CA over the years. When corrected for CA (chronological age), BA/CA and BA-CA did not show a statistical significance between the different study periods. Increasing age and bone age was not influenced by the number of boys included each year. Table 1 presents only girls for consistency (Table 1).

**Table 1:** Demographics of girls.

	Age Dx (years)	BMI (kg/m^2^)	BA (years)	BA/CA	BA-CA (years)
Pre-Covid-19	7.1 ± 2.1 (1.8–10.0)	17.6 ± 2.8 (12.7–25.5)	9.5 ± 2.6 (1.6–13.0)	1.3 ± 0.2 (1.0–2.0)	2.3 ± 1.1 (0–5.0)
Covid-19	7.35 ± 1.40 (1.5–9.8)	18.2 ± 3.5 (11.4–29.4)	9.4 ± 1.7 (2.5–12.3)	1.3 ± 0.2 (0.9–2.0)	1.8 ± 1.1 (−0.7–4.2)
Post-Covid-19	8.01 ± 1.28* (3.1–10.3)	18.5 ± 2.5 (13.6–29.3)	10.4 ± 1.3 years* (6.8–13)	1.27 ± 0.23 (1–1.75)	2.22 ± 1.4 (0–5.7)

Mean ± SD (range), Dx, diagnosis; BMI, body mass index; BA, bone age; CA, chronological age.

* *P* < 0.01 vs. Covid-19 year.

### Days from diagnosis to treatment order and to start of treatment

Average time from diagnosis to GnRHa order was 94.2 ± 109.2 (4.0–397.0) days and average time from GnRHa order to first injection was 54 ± 62 days pre-covid 19. During Covid-19, the average time was 65.70 ± 77.8 (0–306) days between diagnosis and GnRHa order, and 62 ± 65 days between GnRHa order and treatment onset. In the 2 years post-covid the average time from diagnosis to GnRHa order was much shorter (*p* = 0.002) with a range of 33.3 ± 71.5 (0–364) and from order to treatment was similar at 76 ± 72 (14–343) days. The time range is broad for all years (Table 2).

**Table 2:** Time to treatment order and 1st injection.

	Days from Dx to GnRHa order	Days from GnRHa order to 1st treatment
Pre-Covid-19	94.2 ± 109.2 (4.0–397.0)	54 ± 62 (4.0–287.0)
Covid-19	65.70 ± 77.8 (0–306.0)	62 ± 65 (5.0–285.0)
Post-Covid-19	33.4 ± 71.5* (0–364)	76.0 ± 72.0 (14–343)

Mean ± SD (range), Dx, diagnosis.

* *p* < 0.01 vs. Covid-19 year.

### CPP incidence and COVID-19 peaks in the community

**Figure 1:**
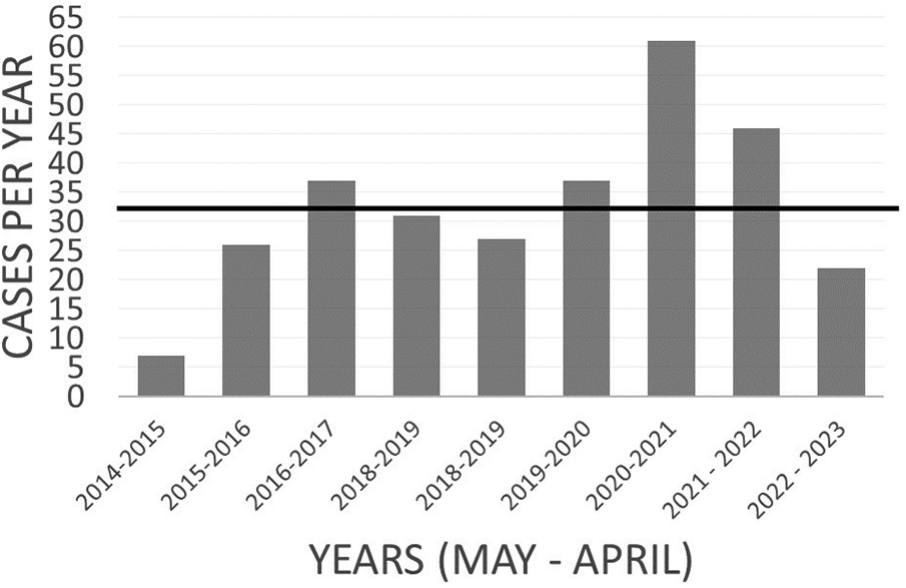
CPP cases by years. Years defined May of one year through April of the following year.

We previously reported the number of cases per month appeared to increase approximately 5–6 months after the highest peak of Covid-19 cases at the time in January 2021, however after an even greater peak in Covid-19 cases in January 2022, there was an apparent rise in CPP cases 6 months later, but to a lesser degree (5 cases in 2022 vs. 11 cases in 2021) although the percent of cases for the year was similar (Figure 1). Thirty two out of 64 cases (50%) were started on treatment in the 3 months between May and July 2021 and 20 of 46 cases (43%) between May and July 2022.

## Discussion

The number of cases of CPP requiring treatment with GnRHa more than doubled during the first year of Covid-19 compared to prior years, and rates dropped again to previous numbers post Covid-19 pandemic.

Time from diagnosis to onset of treatment was similar in the 1st year of Covid-19 compared to pre-Covid-19, but decreased post-covid-19 pandemic. This may be related to increased support from pharmacy technicians at our institution, and potentially, a faster response for authorization from insurance companies. Additionally, time to onset of treatment might have been longer during the pandemic due to higher volume of patients. In our study, BMI was similar for both groups, so the higher incidence of CPP during the pandemic is unlikely to be related to nutrition or weight gain.

Bone age advancement was not different between the Covid-19 pandemic and post-pandemic timeframes, suggesting there was no delay in presentation for medical care during Covid-19 at our institution. In contrast, however, age at diagnosis was significantly higher post-covid, which is an unexpected finding as one would infer access to healthcare was easier during the post-pandemic period, although access at our center didn't seem to be affected based on our data. However, some patients and families might have delayed medical attention during and immediately after the pandemic, thus presenting at an older age after the pandemic resolved. Another consideration for the older age at diagnosis post Covid-19 is the possible trend towards considering treatment of slightly older 7 to 8 year old girls.

We previously reported a trend of increased CPP cases approximately 5–6 months following Covid-19 peaks (1). As we continued to analyze the data overtime, we again observed that CPP cases increased after the largest Covid-19 peak in December, 2021 (data not shown). Our findings are concordant with those reported by Chioma et al. (17), who showed a decline in CPP cases to pre-pandemic numbers after 2021, compared to an increase observed during the Covid-19 pandemic.

In our initial report showing an increase in the incidence of CPP during Covid-19 pandemic (1) we hypothesized reasons for increased cases of CPP during the Covid-19 pandemic, which have also been presented by others, including other hypotheses (2–9, 18, 19). Many of those hypotheses would predict a decrease in CPP cases post-pandemic; these include the impact of emotional changes and increased screen use, sedentarism, nutrition and sleep disturbances seen during the pandemic, which theoretically could have caused an impact on biochemical changes in the brain (implicating gamma aminobutyric acid, N-methyl-D-aspartate, catecholamines, dopamine, and melatonin). The negative psychological impact, more sedentary lifestyle, more time spent indoors, and more prolonged use of electronic devices during the pandemic theoretically changed post-Covid-19 with a return to in-person school and extracurricular activities. Likewise, the direct relationship to the viral infection and the immune response, should all have decreased post-Covid-19. None of the children in the first year of Covid-19 were vaccinated, so the increase in CPP was not related to the vaccine. By the first year post-Covid-19, many children were vaccinated. Since the incidence of CPP decreased during that time, the vaccine is not related to causing CPP. However, the converse cannot be proven. That is, a decrease in cases could be related to the vaccine protecting children or could be unrelated.

Strengths of our study include continued analyses post-Covid-19, which is important as data are scant. The same search criteria were used as in the previous study, so even if the absolute numbers may miss some children evaluated, the relative numbers should be consistent. We used the same months for 4 years to control for any potential seasonal variation. We only report cases of CPP who initiated treatment with GnRHa. We list this primarily as a strength, since this ensures inclusion of only those truly requiring treatment based on all parameters. Another strength of our study is the description of CPP cases in relationship to Covid-19 peaks noted in children in California.

A continued limitation of our study is that we were unable to determine how many children had a confirmed Covid-19 infection. The study was retrospective, but EMR query and potential extraction errors cannot all be assessed.

In conclusion, CPP cases requiring GnRHa treatment significantly increased during the first year of Covid-19 compared to pre-Covid-19 years. Cases started to decrease after the first Covid-19 year, and even more so the following year. The factors that led to a higher incidence of CPP during the pandemic appear to be reversible, but further study with larger cohorts is needed to understand the pathogenic factors contributing to a higher incidence of CPP in association with Covid-19 and whether this trend continues.

## Data Availability

The original contributions presented in the study are included in the article/Supplementary Material, further inquiries can be directed to the corresponding author.
